# Onconephrology: a new frontier in medicine

**DOI:** 10.1590/2175-8239-JBN-2025-E004en

**Published:** 2025-03-03

**Authors:** Carolina Maria Pozzi, Juliana El Ghoz Leme

**Affiliations:** 1 Santa Cruz Hospital, Nephrology Department, Curitiba, PR, Brazil.; 2 Mackenzie Evangelical University Hospital, Renal Transplantation Department, Curitiba, PR, Brazil.

Onconephrology, an emerging discipline in medicine, is dedicated to the study of the complex relationships between neoplasms and kidney diseases. In light of the increasing prevalence of cancer cases worldwide, there is a growing need to deepen research on the epidemiology and pathophysiological mechanisms involved in the intersection of oncology and nephrology^1,2^.

Among the various clinical manifestations of kidney diseases related to neoplasms, glomerulopathies have been receiving attention for approximately 30 years^3^. For a long time, much of the literature has been based on case series, which limits the statistical power in assessing this correlation.

In Brazil, data from INCA (National Cancer Institute) estimate 704,000 new cancer cases annually between 2023 and 2025^4^. Mechanisms potentially involved in the relationship between cancer and glomerulopathies include glomerular disease caused by the neoplasm itself, immunosuppressive therapy used in the treatment of glomerular diseases, which predisposes to neoplasms—especially with prolonged exposure—and treatment of neoplasms with drugs that can induce glomerular injury with clinically evident manifestations^5^.

The challenges faced by nephrologists in the field of oncology undoubtedly encompass glomerulopathies. In these patients, the risk of cancer is up to three times higher compared to the general population, particularly during the period of one year before and one year after a renal biopsy.

Glomerulopathy can precede or follow the diagnosis of cancer, and the role of nephrology care goes beyond treating the kidney disease in these cases. It is worth noting that studies have shown that about half of the patients may have the nephrological manifestation before the oncological diagnosis. Therefore, it is crucial for nephrologists to be familiar with this correlation and remain vigilant for signs and symptoms associated with neoplasms^5,6^.

Although there is no consensus yet on screening criteria for this population, special attention should be given to patients with nephrotic syndrome, especially in cases of membranous glomerulopathy. Emerging biomarkers and new diagnostic tests are promising in differentiating primary from paraneoplastic glomerulopathies, contributing to timely, precise, and effective therapeutic approach^7,8^.

It is extremely important that studies are conducted in local populations, detailing clinical and demographic characteristics, and spreading the knowledge so that nephrologists are equipped to handle cases without overlooking the possibility of a neoplastic diagnosis in patients with glomerulopathies.

In a recent study by Laferreira and Kirsztajn^9^, a Brazilian cohort with a significant number of potentially paraneoplastic glomerulopathies in a large center was evaluated. In this cohort, just over a third of patients with glomerulopathy and neoplasm had a diagnosis of hematological cancer. Among solid tumors, the most prevalent were colon, rectal, and gynecological tumors, which reveals a patient profile requiring closer surveillance during the management of glomerular disease, both in terms of investigating neoplasms at the time of the nephrological diagnosis and maintaining vigilance for neoplasms after the nephrological diagnosis.

In line with other data in the literature, this study found that membranous glomerulopathy was the most frequent condition, and in nearly half of the cases, glomerulopathy was diagnosed before the neoplasm. Among patients who had the oncological diagnosis after, nearly half had their neoplasm identified within one year of renal disease follow-up, which should be considered the period of greatest vigilance. The anti-PLA2r antibody can be an ally in differentiating primary from secondary membranous glomerulopathy, although when positive, it does not completely rule out the possibility of secondary disease.

Studies such as these emphasize the need for careful observation of this patient group, which is essential for enhancing our understanding of the mechanisms involving kidney disease in the oncological context and vice versa. This knowledge is crucial for optimizing the clinical management of these cases and fostering significant advances in care strategies for these populations.
