# Supplementary material for: Onconephrology: a new frontier in medicine
Source: J Bras Nefrol. 2025 Mar 3;47(1):e2025E004. doi: 10.1590/2175-8239-JBN-2025-E004en (PMC11884816; doi:10.1590/2175-8239-JBN-2025-E004en)
Supplement: Translation

# Translation: Onconefrologia: uma nova fronteira na medicina

**DOI:** 10.1590/2175-8239-JBN-2025-E004pt

**Authors:** Carolina Maria Pozzi, Juliana El Ghoz Leme

**Affiliations:** 1 Santa Cruz Hospital, Nephrology Department, Curitiba, PR, Brasil.; 2 Mackenzie Evangelical University Hospital, Renal Transplantation Department, Curitiba, PR, Brasil.

A onconefrologia, uma disciplina emergente na medicina, dedica-se ao estudo das complexas relações entre neoplasias e doenças renais. Diante da crescente prevalência de casos de câncer em todo o mundo, existe uma necessidade cada vez maior de aprofundar a pesquisa sobre a epidemiologia e os mecanismos fisiopatológicos envolvidos na interseção entre oncologia e nefrologia^1,2^.

Dentre as diversas manifestações clínicas de doenças renais relacionadas a neoplasias, as glomerulopatias vêm recebendo destaque há aproximadamente 30 anos^3^. Por um longo período, grande parte da literatura baseou-se em séries de casos, o que limita o poder estatístico na avaliação dessa correlação.

No Brasil, dados do INCA (Instituto Nacional do Câncer) estimam 704.000 novos casos de câncer anualmente entre 2023 e 2025^4^. Os mecanismos potencialmente envolvidos na relação entre câncer e glomerulopatias incluem doença glomerular causada pela própria neoplasia, terapia imunossupressora utilizada no tratamento de doenças glomerulares, que predispõe a neoplasias - especialmente com exposição prolongada - e tratamento de neoplasias com medicamentos capazes de induzir lesão glomerular com manifestações clinicamente evidentes^5^.

Os desafios enfrentados pelos nefrologistas no campo da oncologia, sem dúvida, incluem as glomerulopatias. Nesses pacientes, o risco de câncer é até três vezes maior em comparação com a população geral, especialmente durante o período de um ano antes e um ano após a biópsia renal.

A glomerulopatia pode preceder ou suceder o diagnóstico de câncer, e o papel do cuidado nefrológico nesses casos vai além do tratamento da doença renal. Vale ressaltar que estudos demonstram que cerca de metade dos pacientes pode apresentar a manifestação nefrológica antes do diagnóstico oncológico. Portanto, é fundamental que os nefrologistas estejam familiarizados com essa correlação e permaneçam atentos aos sinais e sintomas associados a neoplasias^5,6^.

Embora ainda não haja consenso sobre os critérios de triagem para essa população, deve-se dar atenção especial aos pacientes com síndrome nefrótica, especialmente nos casos de glomerulopatia membranosa. Biomarcadores emergentes e novos testes diagnósticos são promissores na diferenciação entre glomerulopatias primárias e paraneoplásicas, contribuindo para uma abordagem terapêutica oportuna, precisa e eficaz^7,8^.

É de extrema importância a realização de estudos em populações locais, detalhando características clínicas e demográficas, além da disseminação do conhecimento, de modo a capacitar os nefrologistas para o manejo dos casos sem negligenciar a possibilidade de um diagnóstico neoplásico em pacientes com glomerulopatias.

Em um recente estudo realizado por Laferreira and Kirsztajn^9^, avaliou-se uma coorte brasileira com um número significativo de glomerulopatias potencialmente paraneoplásicas em um grande centro. Nessa coorte, pouco mais de um terço dos pacientes com glomerulopatia e neoplasia apresentava um diagnóstico de câncer hematológico. Dentre os tumores sólidos, os mais prevalentes foram os tumores de cólon, reto e ginecológicos, o que revela um perfil de paciente que requer vigilância mais rigorosa durante o manejo da doença glomerular, tanto em termos de investigação de neoplasias no momento do diagnóstico nefrológico quanto na manutenção da vigilância para neoplasias após o diagnóstico nefrológico.

Em consonância com outros dados da literatura, este estudo constatou que a glomerulopatia membranosa foi a condição mais frequente e, em quase metade dos casos, foi diagnosticada antes da neoplasia. Entre os pacientes cujo diagnóstico oncológico foi feito posteriormente, quase metade teve sua neoplasia identificada dentro de um ano de acompanhamento da doença renal, período que deve ser considerado o de maior vigilância. O anticorpo anti-PLA2r pode ser um aliado na diferenciação entre glomerulopatia membranosa primária e secundária, embora, quando positivo, não exclua completamente a possibilidade de doença secundária.

Estudos como esse enfatizam a necessidade de observação cuidadosa desse grupo de pacientes, essencial para aprimorar nossa compreensão dos mecanismos que envolvem a doença renal no contexto oncológico e vice-versa. Esse conhecimento é fundamental para otimizar o manejo clínico desses casos e promover avanços significativos nas estratégias de cuidado para essas populações.
